# Buckwheat (*Fagopyrum esculentum*) Hulls Are a Rich Source of Fermentable Dietary Fibre and Bioactive Phytochemicals

**DOI:** 10.3390/ijms242216310

**Published:** 2023-11-14

**Authors:** Zhihong Zhang, Songtao Fan, Gary J. Duncan, Amanda Morris, Donna Henderson, Philip Morrice, Wendy R. Russell, Sylvia H. Duncan, Madalina Neacsu

**Affiliations:** 1 School of Grain Science and Technology, Jiangsu University of Science and Technology, Zhenjiang 212100, China; zhangzhihong0415@just.edu.cn; 2 Rowett Institute, University of Aberdeen, Foresterhill, Aberdeen AB25 2ZD, Scotland, UK; fansongtao@ujs.edu.cn (S.F.); gary.duncan@abdn.ac.uk (G.J.D.); a.morris@abdn.ac.uk (A.M.); d.henderson@abdn.ac.uk (D.H.); rwt032@abdn.ac.uk (P.M.); w.russell@abdn.ac.uk (W.R.R.); sylvia.duncan@abdn.ac.uk (S.H.D.); 3 School of Food and Biological Engineering, Jiangsu University, Zhenjiang 212013, China

**Keywords:** buckwheat (*Fagopyrum esculentum*) hulls, phytochemicals, microbial metabolites, enzyme bioprocessing, in vitro digestion, in vitro fermentation, short chain fatty acids, dietary fibre, gut microbiota

## Abstract

Pseudo-cereals such as buckwheat (*Fagopyrum esculentum*) are valid candidates to promote diet biodiversity and nutrition security in an era of global climate change. Buckwheat hulls (BHs) are currently an unexplored source of dietary fibre and bioactive phytochemicals. This study assessed the effects of several bioprocessing treatments (using enzymes, yeast, and combinations of both) on BHs’ nutrient and phytochemical content, their digestion and metabolism in vitro (using a gastrointestinal digestion model and mixed microbiota from human faeces). The metabolites were measured using targeted LC-MS/MS and GC analysis and 16S rRNA gene sequencing was used to detect the impact on microbiota composition. BHs are rich in insoluble fibre (31.09 ± 0.22% as non-starch polysaccharides), protocatechuic acid (390.71 ± 31.72 mg/kg), and syringaresinol (125.60 ± 6.76 mg/kg). The bioprocessing treatments significantly increased the extractability of gallic acid, vanillic acid, p-hydroxybenzoic acid, syringic acid, vanillin, syringaldehyde, p-coumaric acid, ferulic acid, caffeic acid, and syringaresinol in the alkaline-labile bound form, suggesting the bioaccessibility of these phytochemicals to the colon. Furthermore, one of the treatments, EC_2 treatment, increased significantly the in vitro upper gastrointestinal release of bioactive phytochemicals, especially for protocatechuic acid (*p* < 0.01). The BH fibre was fermentable, promoting the formation mainly of propionate and, to a lesser extent, butyrate formation. The EM_1 and EC_2 treatments effectively increased the content of insoluble fibre but had no effect on dietary fibre fermentation (*p* > 0.05). These findings promote the use of buckwheat hulls as a source of dietary fibre and phytochemicals to help meet dietary recommendations and needs.

## 1. Introduction

Climate change is likely to result in huge crop losses [1,2] and represents a global challenge in delivering food and nutrition security. As such, the staple crops that currently provide a dominant proportion of global food include maize (*Zea mays*), wheat *(Triticum aestivum*), and rice (*Oryza sativa*) which in the future may not withstand extreme weather events both predicted and now being increasingly seen [3,4]. As a consequence of focusing on the big three crops, other underutilised or largely neglected crops have been in decline, both commercially and agriculturally, but have the potential to contribute to diversifying cropping systems and food sources [5]. Agriculture worldwide needs to undergo significant adjustment in response to climate change to contribute to sustainable farming and food supply, calling for a more diversified agriculture, adopting novel and/or underutilised crops including pseudo-cereals, such as buckwheat [2,5].

Worldwide, only residents in a few countries consume enough fibre to reach or exceed the daily fibre recommendation of 30 g of fibre per day as recommended by WHO [6]. Emerging research evidence shows that dietary changes from a diet rich in fibre to a diet low in fibre (and high in saturated fats) directly contribute to the development of obesity, metabolic syndrome, and cardiovascular diseases [7,8]. Pseudo-cereals, such as buckwheat, are a rich source of fibre and bioactive phytochemicals, particularly polyphenols, anthocyanins, and flavonoids, providing health benefits, including having cardioprotective effects and stimulating insulin secretion in diabetes mellitus type 2 [2,9,10]. Together with fibre and phenolic compounds, buckwheat is a great source of bioactive peptides [11].

Buckwheat is an underutilised crop which offers great opportunities to expand diversity and implement robust agro-industrial systems to address climate change. It is estimated that buckwheat production is around 1.9 million tons globally and could offer an important dietary energy source for humans [12]. During the buckwheat grain milling process, approximately 17% of hull products are discarded resulting in a large amount of waste material [13,14]. The outer layer (bran and hull) and germ fraction of buckwheat are rich in phytochemicals [15] with phenolic compounds reaching 330 mg/100 g dry matter in buckwheat hulls (BHs), much higher than that found in other flours [16]. However, only 5–10% of the phenolics can be absorbed by the small intestine, and the remaining 90–95% unabsorbed phenolics are transported to the large intestine and metabolised by the gut microbiota [17], with gut microbes affecting the conversion of phenolics and the phenolics metabolites influencing the gut bacterial composition [18,19,20]. There is a growing amount of research dealing with in vitro or in vivo models to elucidate the metabolism and health benefits of nutrients and phytochemicals; however, the complex microbial metabolisms and transformations in the human colon remain unclear [21]. 

**Figure 1:**
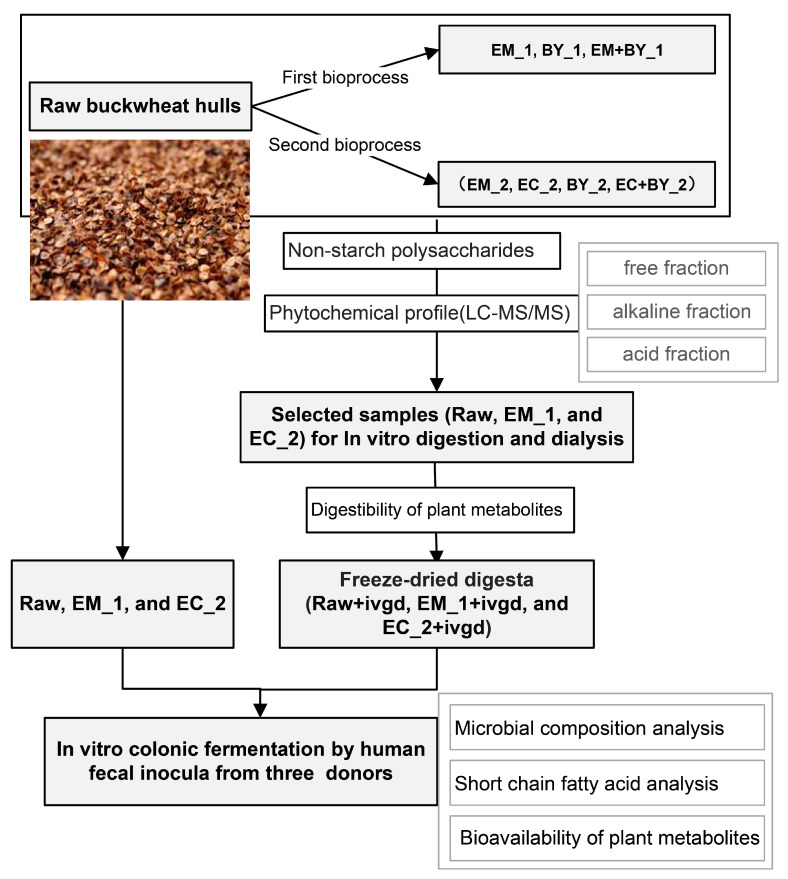
Overview of experimental design: The bioprocessing treatments used in enzyme mixture I (EM), enzyme mixture II (EC), yeast fermentation (BY), and combinational treatments. The plant metabolites of the raw, first-bioprocessed, and second-bioprocessed buckwheat hulls were extracted into their free and bound forms and analysed by targeted LC-MS/MS; the monosaccharide compositions of soluble and insoluble non-starch polysaccharides were analysed with gas chromatography. The EM_1 (enzymes mixture I) and EC_2 (enzymes mixture II) treatments were selected for in vitro digestion and dialysis system (IVDG) and for in vitro fermentation studies. The concentrations of short chain fatty acids and other digestion and fermentation metabolites were measured using GC and LC-MS/MS analyses, respectively. The microbial composition analysis was performed with 16S rRNA gene sequencing.

With the aim of increasing the sustainability of food production by reducing the by-products generated while delivering a circular nutrition, this study promotes buckwheat hulls as a novel fibre- and phytochemical-rich food ingredient by assessing its nutrients’ and chemicals’ in vitro digestion and microbial metabolism (see Figure 1). 

## 2. Results

### 2.1. Buckwheat Hulls Composition

#### Buckwheat Hulls Are a Rich Source of Insoluble Dietary Fibre (Non-Starch Polysacchrides) (NSPs)

The nutrient composition of BHs is shown in Table 1. BHs have a low (2.5%) total starch, with undetectable levels of resistant starch and low levels of protein (4.05%) and fat (0.13%). More than 30% of their composition is insoluble NSPs (non-starch polysaccharides). Total amino acids in BHs are 25.67 mg/g, consisting of essential amino acids (EAAs, 1.04%), and non-essential amino acids (non-EAAs,1.53%), covering all the individual EAAs (Table 1). The three most abundant individual amino acids are non-EAA glutamic acid (0.33%), aspartic acid (0.25%), and cysteic acid (0.23%), and the content of individual EAAs was less than 2 mg/g. 

### 2.2. Bioprocessing of the Buckwheat Hulls 

#### 2.2.1. Bioprocessing Treatments of Buckwheat Hulls Promote Increases in NSP Content

Monosaccharide composition analysis of NSPs (Table 2) indicated that insoluble fibre in the BHs was mainly composed of 15.78% xylose, 9.67% glucose, and 3.68% uronic acid, which suggested the existence of a hemicellulose rich-fraction (such as xylan, xyloglucan, arabinoxylan, or galactoxyloglucan) along with pectin-type polysaccharides. The soluble fibre in the BHs was also mainly composed of 0.06% xylose, 0.09% glucose, and 0.09% uronic acid. Thus, xyloglucans derivates with uronic acid are probably the main fibre type in raw BHs. 

Both bioprocessing treatments consistently increased the detectability of the content of insoluble NSPs to different degrees, while slightly affecting the content of soluble NSPs (Table 2). Specifically, the first bioprocessing treatments increased the content of soluble NSPs while the second bioprocessing treatments except BY_2 decreased the content of soluble NSPs. For the first bioprocessing treatment groups, the highest content of insoluble NSPs was observed in EM_1 (46.41%), followed by BY_1 (42.60%), and EM+BY_1 (40.09%). Meanwhile, the highest soluble NSP content was obtained in BY_1 group (0.54%), followed by EM+BY_1 (0.34%) and EM_1 (0.31%). After the second bioprocessing treatments using EM_2, the highest content of insoluble NSPs (37.68%) was observed, followed by EC_2 (37.43%) and EC+BY_2 (35.64%). Meanwhile, after treatment with EM_2, the lowest content of soluble NSPs (0.12%) was obtained, followed by EC+BY_2 (0.15%) and EC_2 (0.18%). These results indicated that both the first and second bioprocessing treatments contributed to the detectability of the insoluble NSPs, and the first bioprocessing treatments are more effective than the second bioprocessing treatments. As for the monosaccharides composition of insoluble NSPs, both the first and second bioprocessing regimes consistently increased the content of xylose, glucose, arabinose, galactose, rhamnose, and uronic acid. The highest content of insoluble xylose was observed in EM_2 (20.51%), followed by EC_2 (20.37%) and EM_1 (19.73%). As for the glucose, the highest content was measured following the first bioprocessing treatments. 

#### 2.2.2. Bioprocessing Treatments of Buckwheat Hulls Increased the Extractability of Several Plant Metabolites

**Figure 2:**
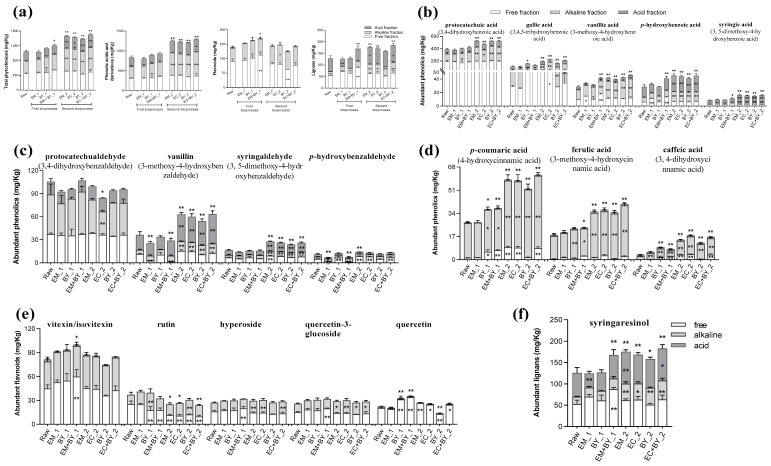
Total content of plant metabolites in the free, alkaline-labile, and acid-bound forms of raw and bioprocessed buckwheat hulls, including total phytochemicals, phenolic acids and derivatives, flavonoids, and lignans (**a**). Total content is the cumulative sum of the content of individual plant metabolites measured LC-MS/MS in free form, alkaline form, or acid form. Individual phenolic acids and derivatives with a total content of free, alkaline, and acid forms of more than 10 mg/kg affected by both first and second bioprocessing technique, including derivatives of benzoic acid (**b**), benzaldehydes (**c**), and cinnamic acids (**d**). Individual flavonoids with a total content of free, alkaline, and acid forms of more than 10 mg/kg (**e**). The most abundant lignan compound (**f**). Within the first bioprocess: EM_1 = enzymes mixture I, BY_1 = Baker’s Yeast, EM+BY_1 = Baker’s yeast fermentation together with enzymes mixture I, within the second bioprocess: EM_2 = enzymes mixture I, EC_2 = enzymes mixture I with cellulase, BY_2 = Baker’s Yeast, EC+BY_2 = Baker’s yeast fermentation together with mixture I and cellulose. Data are represented with the mean ± S.D. Asterisks indicate the significance between the raw and bioprocessed samples, using One-Way ANOVA test: (*) *p* < 0.05 and (**) *p* < 0.01.

Quantitative profiling of individual plant metabolites in BHs by targeted LC-MS/MS is shown in Figure 2a–f, including phenolic acids and derivatives (PADs), flavonoids, and lignans. The total content of the abovementioned three type compounds is defined as the sum of the free, alkaline-labile, and acid-labile fractions of each type. Generally, BHs are also a rich source of plant metabolites with a total content of 1167.45 mg/kg, summing PADs (831.40 mg/kg), flavonoids (208.93 mg/kg), and lignans (127.11 mg/kg) of 129 molecules measured. The bioprocessing treatments showed various effects on the extractability of these compounds, with the highest total content being 1693.13 mg/kg after EC+BY_2 treatment. Other bioprocessing treatments with significant impact on the extractability of phenolics molecules were EM+BY_1 with 1355.82 mg/kg (*p* < 0.05), EM_2 with 1647.48 mg/kg (*p* < 0.01), EC_2 with 1616.30 mg/kg (*p* < 0.01), and BY_2 with 1559.57 mg/kg (*p* < 0.01).

Compared with the PADs of raw BHs (831.40 mg/kg), both the first and second bioprocessing steps increased the total content except for the EM_1 treatment, with significant differences observed in the second bioprocessing groups (*p* < 0.01) (Figure S1). Notably, the second bioprocessing significantly increased the content of alkaline-labile PADs, contributing to a substantial increment of approximately 50% in the total content. In terms of the flavonoids of the raw BHs (208.93 mg/kg), the first bioprocessing slightly increased the total content while the second bioprocessing decreased the amount of flavonoids compared to the raw BHs. The EM+BY_1 treatment increased the extractability of the phytochemicals into the free fraction contributing to the total chemical extractability, while BY_2 had an opposite effect. The second bioprocessing significantly increased the release of the lignans into the alkaline-bound fraction, contributing to the increment in the total lignan content (*p* < 0.01 or 0.05). The EM_1 and EM_2 treatments significantly increased the content of lignans released into the acid-labile fraction (*p* < 0.01 and *p* < 0.05, respectively). In addition, EM+BY_1 maximally increased the extractability of lignans in the free fraction, resulting in an increase in the total content (*p* < 0.01) while EC+BY_2 resulted in a significant increase in the acid-bound fraction (*p* < 0.05). The results showed that the second bioprocessing promoted the chemical extractability of alkaline-bound plant metabolites release while the first bioprocessing was less effective in promoting this release. 

Figure 2b–d show several PADs with a total higher content of 10 mg/kg, including derivatives of benzoic acid (Figure 2b), benzaldehydes (Figure 2c), and cinnamic acids (Figure 2d). Among the derivatives of benzoic acid in the raw BHs, protocatechuic acid (390.71 mg/kg), gallic acid (108.61 mg/kg), protocatechualdehyde (105.64 mg/kg), vanillin (36.32 mg/kg), p-hydroxybenzoic acid (28.51 mg/kg), vanillic acid (28.39 mg/kg), p-coumaric acid (27.29 mg/kg), ferulic acid (18.25 mg/kg), syringaldehyde (16.28 mg/kg), p-hydroxybenzaldehyde (11.11 mg/kg), syringic acid (8.81 mg/kg), and caffeic acid (3.68 mg/kg) were the most abundant metabolites. A full list of the detected plant metabolites is presented in Supplementary Table S1. A significant increase in the extractability of alkaline-bound forms was observed among these PADs after the second bioprocessing, except for the protocatechualdehyde and p-hydroxybenzaldehyde (Figure 2). In addition, the second bioprocessing treatments increased the extractability of *p*-hydroxybenzoic acid, syringic acid, vanillin, and syringaldehyde in the acid-labile fraction. The first bioprocessing mainly increased the extractability of the free and alkaline-bound fractions of the cinnamic acid derivatives except for the *p*-coumaric acid after the EM_1 treatment. 

The flavonoids (Figure 2a,e) were abundant in the raw BHs summing 207.75 mg/kg, with 132.45 mg/kg of flavonoids all extractable in free fraction, followed by the alkaline-bound fraction (70.30 mg/kg), and acid-bound fraction (5.00 mg/kg). The vitexin (and isovitexin) was the richest flavonoid measured in raw BHs, with a total content of 81.53 mg/kg, mainly extracted in the free fraction (45.00 mg/kg), followed by alkaline-bound (33.65 mg/kg), and acid-bound fractions (2.88 mg/kg). The first bioprocessing increased the extractability of vitexin in the free fraction while the second bioprocessing treatments showed limited effect on the extractability of vitexin. In the BHs, rutin (37.10 mg/kg) was extracted only in the free (25.15 mg/kg) and alkaline-bound (11.94 mg/kg) fractions. Both the first and second bioprocessing treatments significantly decreased the extractability of rutin in the free fraction (*p* < 0.01, with the exception of EM_1 treatment) and increased the extractability in the alkaline-bound fraction with significant differences observed in BY_1 and BY_2 (*p* < 0.01). Syringaresinol accounted for 98.81% of the lignans measured, with a total content of 125.60 mg/kg (52.55 mg/kg being extracted in free fraction and 73.05 mg/kg in the bound form). The effect of bioprocessing on syringaresinol levels resulted in a significant increase in its extractability in the bound fractions of PADs and lignans, and had limited impact on its extractability in the free fraction.

#### 2.2.3. Bioprocessing of Buckwheat Hulls Improved the Release of Plant Metabolites in the Upper Gastrointestinal Tract during In Vitro Digestion

Based on the results presented so far, the EM_1 and EC_2 treatments were selected to further investigate their effect on in vitro digestion and microbial metabolism of BHs. For this, an in vitro digestion model named IVGD was used to study the metabolism and bioavailability of BH components. In Table 3 are presented the metabolites resulting from IVGD of the raw and bioprocessed BHs. 

The most abundant metabolites measured after the in vitro digestion such as protocatechuic acid, protocatechualdehyde, vanillin, p-hydroxybenzoic acid, 3, 5-dimethoxy-4-hydroxybenzaldehyde, and vanillic acid, were also highly extractable in both free and alkaline-bound forms (suggesting, therefore, high in vitro upper GI bioaccessibility). Similarly, the metabolites which had a moderate or low extractability in the free fraction were found to have moderate or low in vitro upper GI bioaccessibility, specifically p-hydroxybenzaldehyd, salicylic acid, chlorogenic acid, chlorogenic acid, 3, 5-dimethoxy-4-hydroxybenzoic acid, 3-hydroxymandelic acid, 2-hydroxybenzyl alcohol, p-coumaric acid, phenylacetic acid, ferulic acid, 3, 4-dihydroxyphenylacetic acid, 4-hydroxyphenylacetic acid, caffeic acid, and 2, 3-dihydroxybenzoic acid. This was with the exception of the 3, 4, 5-trihydroxybenzoic acid, which mainly was extracted in free and alkaline-bound forms and also had low in vitro bioaccessibility. The EM_1 and EC_2 treatments significantly and consistently increased the in vitro bioaccessibility of vanillin, p-hydroxybenzoic acid, p-hydroxybenzaldehyde, salicylic acid, 3-hydroxymandelic acid, p-coumaric acid, and ferulic acid (Table 3). The EC_2 treatment specifically increased the bioaccessibility of protocatechuic acid, protocatechualdehyde, vanillic acid, 3, 4, 5-trihydroxybenzoic acid, and 3, 4-dihydroxyphenylacetic acid. Overall, EC_2 treatment was more efficient in increasing the in vitro upper gastrointestinal release of the plant metabolites from BHs in comparison with the EM_1 treatment. 

#### 2.2.4. The Main Microbial Metabolism Transformation of Buckwheat Hulls Components Occurs within the First 24 h

For this study, in vitro anaerobic fermentation using mixed microbiota (from human faeces) from three healthy human volunteers was performed to evaluate the microbial metabolism transformation of main BH components (plant metabolites and nutrients) in the native (raw) material, after the EM_1 and EC_2 treatments and following their in vitro digestions. 

**Figure 3:**
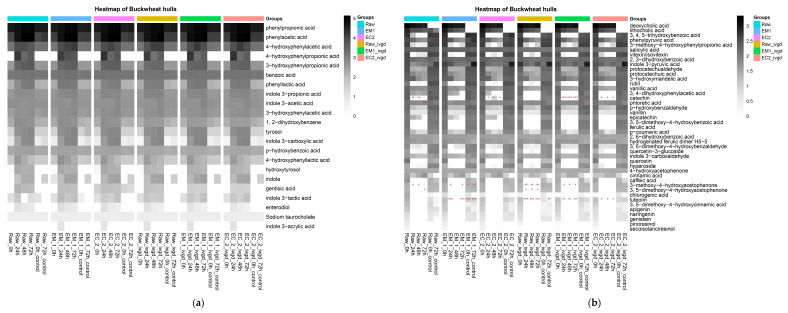

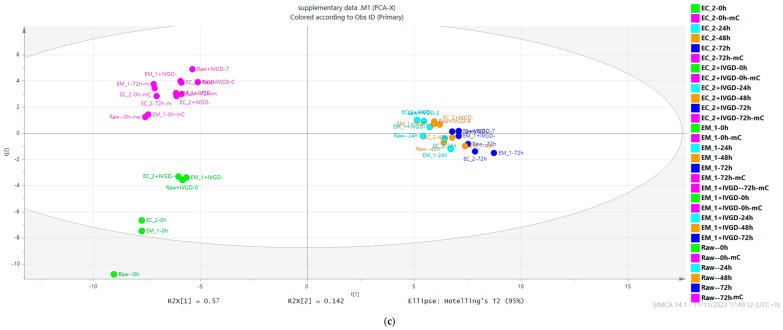
Heatmap showing the changes of plant metabolites from buckwheat hulls before (raw) and after bioprocesses during mixed microbiota incubations, including metabolites with consistent increase (**a**) and consistent decrease (**b**) during the in vitro colonic fermentation. Values were the mean of three donors, and each donor had three replicates. The concentration (log 10) was indicated by a colour gradient, where the darker represents the higher concentration. The multiple *t*-test with the Holm–Sidak method correction was used for statistical comparisons. Significance differences were * *p* < 0.05, ** *p* < 0.01, *** *p* < 0.001 when compared with 0 h samples. Between three donors, the consistently increased and decreased metabolites were marked with red and green, respectively. Principal component analysis (PCA) (unit variance (UV)-scaled) of average microbial metabolites measured from faecal incubations from three human donors (**c**) with baseline samples (0 h in green colour) for Raw, EM_1, and EC_2 treatments-EM and EC, IVDG-predigested samples, their blanks-B (0 and 72 h in pink colour); the metabolites measured at 24 h (bright blue colour), the microbial metabolites measured at 48 h (orange colour), and 72 h (dark blue colour).

A total of 135 compounds were quantified using targeted LC-MS/MS analysis during the microbial fermentation process. Principal Component Analysis (PCA) was used to analyse the distribution of these metabolites, generating the effective visual representations for BH samples. The PCA (Figure 3c) plots depict the grouping of all the control samples (in the upper-left panel), the BH matrix before fermentation (in the lower-left panel), and fermented BH matrix (in the middle-right panel) indicating the significant differences of metabolic profiles among these samples. In the upper-left panel of the PCA plot, the close location of these control samples suggested a stable fermentation process with no significant microbial transformation after 24 h up to the 72 h end point (Figure 3c). In the lower-left panel of the PCA plot, the relatively scattered plots, including raw and enzyme-treated BHs, confirmed the difference induced by bioprocessing treatment on the BH matrix, while the quite close plots of their IVGD samples revealed that IVGD eliminated these differences. Furthermore, the fermented sample located in the middle-right panel demonstrated that all the raw, bioprocessed, and IVGD BHs were distinguished from the unfermented matrix, suggesting a rapid metabolism with a clear and significant change in metabolite profiles within 24 h, and further microbial processing had limited impact on the metabolite profile. In general, these results indicated the bioprocessed and IVGD-treated samples showed little difference in the metabolite profiles of the fermentation process. These metabolite transformations occurred in the first 24 h (Figure 3c) as the metabolite profiles of 24 h (light blue), 48 h (orange), and 72 h (dark blue) were quite similar.

Furthermore, heatmap analysis was used to visualise the temporal change in content of the individual metabolites during the fermentation process. The metabolites with consistent increases across the six matrixes when compared with 0 h are presented in Figure 3a and those with a decrease are presented in Figure 3b. 

The metabolites showing a consistent increase across three donors and six matrixes during in vitro fermentation were mainly the microbial metabolites derived from protein, including the derivatives of aromatic amino acids, tryptophan, tyrosine, and phenylalanine, and some derivatives of ferulic acid and lignans. Specifically, a consistent increase in tryptophan microbial metabolites such as indole, indole 3-propionic acid, indole 3-acetic acid, indole 3-carboxylic acid, indole 3-lactic acid, and indole 3-acrylic acid, were observed; and also the increase in phenylalanine microbial metabolites such as phenylpropionic acid, phenylacetic acid, benzoic acid, phenyllactic acid, and gentisic acid; and the increase in tryrosine microbial metabolites such as 4-hydroxyphenylacetic acid, 4-hydroxyphenylpropionic acid, 4-hydroxybenzoic acid, and 4-hydroxyphenyllactic acid; and the increase in syringaresinol metabolites enterodiol, as well as the ferulic acid microbial metabolite 3-hydroxy phenyl propionic acid. As shown in Figure 3b, the parent compounds referring to the abundant PADs in Figure 2b–d rapidly decreased after microbial transformation. In addition, the compounds and metabolites detected were mostly derivatives of the benzoic, cinnamic, phenylacetic, and phenylpropionic acids. Significant decreases across three donors were observed for catechin in Raw, EC_2, EM_1+IVGD, and EC_2+IVGD; for 3-methoxy-4-hydroxyacetophenone in Raw, EM_1 and their IVGD samples; for 3, 5-dimethoxy-4-hydroxyacetophenone in Raw+IVGD; and for luteolin in EM_1, and Raw+IVGD, EM_1+IVGD, and EC_2+IVG. Generally, this metabolite profile is very representative of people consuming a high fibre and plant protein diet, suggesting that BHs could represent a potential source of nutrients for biodiversification of the human diet and help meet and diversify dietary recommendations.

#### 2.2.5. Buckwheat Hulls Are Fermentable Sources of Dietary Fibre

**Figure 4:**
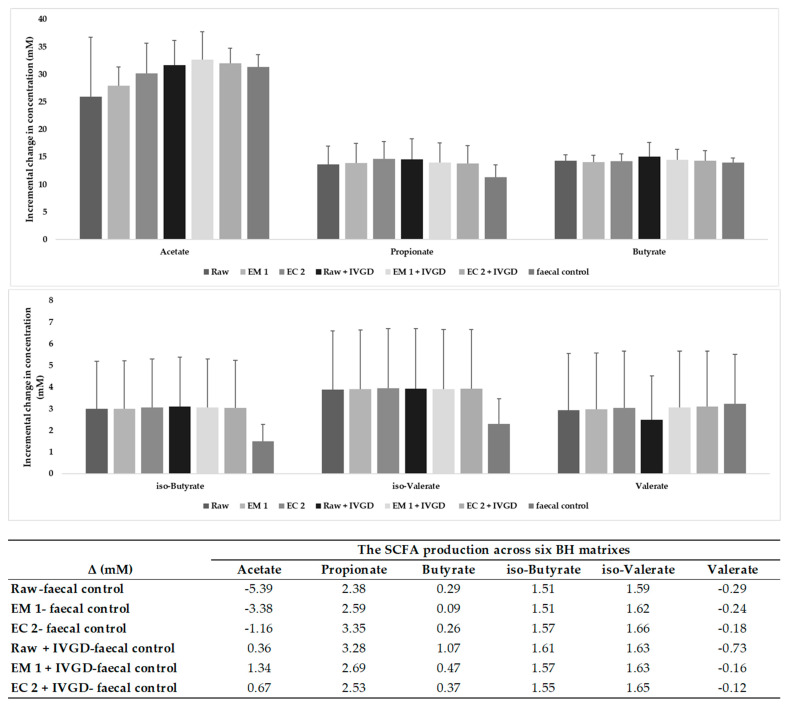
Short chain fatty acids (SCFAs) production over 72 h fermentation as incremental change (mM) in concentration, average ± SD (n = 3). The raw and enzyme-treated buckwheat hulls (EM_1 and EC_2), as well as their IVDG-predigested samples, were inoculated with faecal slurries from each of the 3 donors (donor 1, 2, and 3) and the faecal control samples representing the samples without the BH matrix. Values were calculated by subtracting the value of 0 h from the value of the fermented sample at 72 h for each sample. The SCFAs’ production across six matrixes were estimated by subtraction of the average values of faecal control samples from the average values of incremental changes in SCFA production for each matrix (values underneath plot).

Colonic fermentation of buckwheat hulls’ dietary fibre resulted in the increase in production of short chain fatty acids (SCFAs), including the major SCFAs, mainly propionate, followed by butyrate, and minor SCFAs such as iso-butyrate and iso-valerate. The incremental changes in SCFAs’ production were calculated by subtracting the value of 0 h from the value of the fermented sample at 72 h for each sample (Figure 4 and Figure S2). The SCFAs’ production across six matrixes were estimated by subtraction of the average values of faecal control samples from the average values of incremental changes in SCFA productions for each matrix (see Figure 4 values underneath plot). These results suggest that BH samples are sources of fermentable fibre, promoting the formation mainly of propionate and, to a lesser extent, butyrate formation. The fermentation of dietary fibre from BH matrixes leads to the formation of iso-butyrate and, respectively, to iso-valerate formation. The results showed that bioprocessing and in vitro digestion had limited impact on SCFA production. Therefore, buckwheat hulls could serve as a dietary source of fibre given their fermentability by mixed microbiota and production of SCFAs.

#### 2.2.6. Variations in Bacterial Composition at the Genus and OTU Levels Occur in a Donor-Dependent Manner in Response to Buckwheat Hull Metabolism

**Figure 5:**
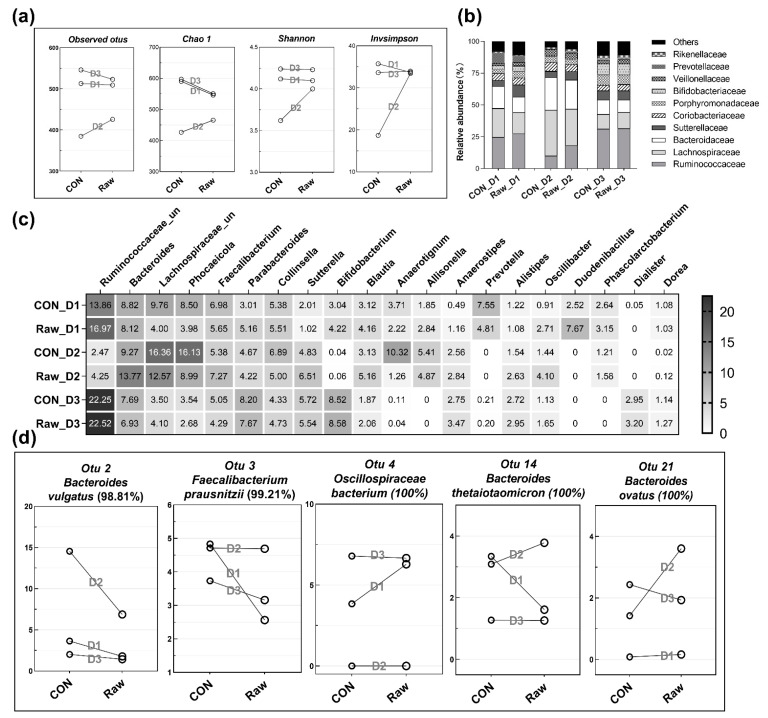
Changes in microbial communities derived from human faecal inocula with raw buckwheat hulls as the sole added energy source or blank medium at 72 h time points. Four different measures of diversity (observed OTU richness, Chao estimate of total richness, Shannon diversity index, and inverse Simpson diversity index) are shown in (**a**). The relative abundance of top 10 bacterial families (**b**). Heatmap of the relative abundance of top 20 bacterial genera, the colour gradient (white–black) indicates the relative abundance of genus as labelled in each square (**c**). The abundance of selected species with sequence identity (**d**). D1, D2, D3 mean donor 1, donor 2, donor 3, respectively.

Changes in microbial community diversity were assessed by calculating observed OTU (operational taxonomic unit) richness, Chao 1 richness, and the Shannon and inverse Simpson diversity indices among three donors (Figure 5a). The addition of raw buckwheat hulls consistently increased the richness and diversity indices in the mixed microbiota from 1 donor (donor 2), but decreased the richness indices and had slight impacts on the diversity indices in donor 1 and 3. Human faecal microbiota is generally dominated by two major phyla, Firmicutes and Bacteriodetes. As for the microbial composition at the family level (Figure 5b), Ruminococcaceae, Lachnospiraceae, and Bacteroidaceae were the three most abundant families accounting for more than 50% of bacterial members for all three donors, and the relative abundance of each of the three families varied among the three donors. For both the control (only culture medium) and fermented groups, Lachnospiraceae was the most abundant family in donor 2, and Ruminococcaceae was the most abundant family in donor 1 and 3. In particular, the addition of raw buckwheat hulls increased the relative abundance of Ruminococcaceae from 10.14% to 18.38% in donor 2. Overall, the addition of raw buckwheat hulls slightly changed the bacterial family composition for all three donors.

At the genus level (Figure 5c), the relative abundances of the 20 most abundant genera displayed donor-dependent differences between two groups. For donor 1, the addition of raw buckwheat hulls greatly increased the abundance of the genera Ruminococcaceae_unclassified (from 13.86% to 16.97%) and Duodenibacillus (from 2.52% to 7.67%), as well as genera Parabacteroides, Bifidobacterium, Blautia, and Oscillibacter to a lesser extent, along with the decreased abundance of Lachnospiraceae_unclassified (from 9.76% to 4.00%), Phocaeicola (from 8.50% to 3.98%), and Prevotella (from 7.55% to 4.81%). For donor 2, the inclusion of raw buckwheat hulls mainly increased the amount of Bacteroides (from 9.27% to 13.77%), Faecalibacterium (from 5.38% to 7.27%), Blautia (from 3.13% to 5.16%), Ruminococcaceae_unclassified (from 2.47% to 4.25%), and Oscillibacter (from 1.44% to 4.10%), and reduced the abundance of Lachnospiraceae_unclassified (from 16.36% to 12.57%), Phocaeicola (from 16.13% to 8.99%), and Anaerotignum (from 10.32% to 1.26%). For donor 3, adding raw buckwheat hulls seemingly had negligible impacts on the relative abundance of these abundant genera. 

To assign species-level classifications, the representative sequences for each OTU were searched against the NCBI Nucleotide database to obtain the sequence similarity value. Similarly, the bacterial composition at the OTU/species level also revealed donor-dependent variations (Figure 5d and Table S2). Among the abundant Bacteroides species, the addition of raw buckwheat hulls greatly decreased the amount of B. vulgatus (OTU 2, 98.81%) in donor 2, and to a lesser extent in donor 1 and 3. In addition, it specifically stimulated the growth of B. thetaiotaomicron (OTU 14, 100%) and B. ovatus (OTU 21, 100%) in donor 2, which were reduced in abundance in donor 1 and donor 3. In comparison, for the abundant Parabacteroides species, the relative abundances of P. distasonis (OTU 9, 100%) and P. merdae (OTU 10, 100%) were merely impacted by the addition of raw buckwheat hulls (Table S2). In addition, raw buckwheat hulls greatly decreased the abundance of Faecalibacterium prausnitzii (OTU 3, 99.21%) in both donor 1 and 3, while increasing the abundance of F. prausnitzii (OTU 27, 96.44) in donor 2 (Table S2). As the most abundant species in Ruminococcaceae_unclassified genus, Oscillospiraceae bacterium (OTU 4, 100%) was specifically enriched by raw buckwheat hulls in donor 2. 

## 3. Discussion

This study investigated the nutritional composition of BHs, finding that they are a rich source of dietary fibre. Buckwheat is reported to be a popular high-protein crop and offers an appreciable level of amino acids [22]. Our results show BHs include all types of the essential amino acids (EAAs). Leucine is an abundant EAA in BHs, acting as a strong insulin secretagogue, playing an important role in inhibiting muscle protein breakdown, and/or promoting muscle protein synthesis in both body health and disease conditions [23]. In terms of dietary fibre, insoluble NSPs (with a 31.09% content) are the predominant fibre in BHs, comprising more than 99% of the total NSPs. In comparison, the flours have approximatively 7% insoluble fibre, and bran without hull fragments has 16% total dietary fibre, of which 75% is of the soluble type [24,25]. These results suggested that BHs could serve as a potential source for dietary fibre. Further, the in vitro faecal fermentation investigation revealed that BH fibre is fermentable, producing high concentrations of SCFAs, mainly propionate. Moreover, there is no obvious difference in SCFA content between the raw and bioprocessed BHs, suggesting no overall impact of bioprocessing on SCFA production after the faecal fermentation of BHs. The bioprocessed BHs still remained fermentable. These results support the use of BHs as a good candidate for dietary fibre biodiversification and in helping to boost dietary fibre intake.

BHs are also a rich source of bioactive phytochemicals [4]. Among those compounds, protocatechuic acid (390.71 mg/kg) was the most abundant plant metabolite measured in the BHs, extracted in the free form (184.81 mg/kg), and bound form (205.91 mg/kg). The in vitro upper gastrointestinal digestion model was applied to evaluate upper gastrointestinal bioaccessibility. The presence of protocatechuic acid in high concentration (300.18 mg/kg) in the dialysate, suggests its high bioaccessibility to the upper gastrointestinal tract. The first bioprocessing process showed no overall impact on the chemical extractability of protocatechuic acid while the second bioprocessing process significantly increased the alkaline-labile content (*p* < 0.05 or 0.01), contributing to a significant increase in the total content (*p* < 0.01). Moreover, different bioprocessing of soluble NSP-rich brans with fermentation or enzymatic and fermentation treatments efficiently improved the in vitro bioaccessibility of phytochemicals [26]. As predicted, the dialysable fraction after EM_1 (268.99 mg/kg) showed a small decrease while, after EC_2 processing (346.67 mg/kg), it displayed a significant increase (*p* < 0.01). Overall, protocatechuic acid following all the treatments showed very good bioaccessibility to the upper gastrointestinal tract. Protocatechuic acid was reported to have strong oxidative properties and is widely used in various ailments associated with oxidative stress damage in multiple body systems. Therefore, dietary intake of protocatechuic acid could be a useful treatment and/or prophylaxis related to oxidative stress damage, such as cancer, inflammation, etc. 

The bioprocessing treatments significantly increased the extractability of several PADs (gallic acid, vanillic acid, *p*-hydroxybenzoic acid, syringic acid, vanillin, syringaldehyde, *p*-coumaric acid, ferulic acid, and caffeic acid), and lignan (syringaresinol) in the alkaline-labile bound form, suggesting a possible release/bioaccessibility of these phytochemicals to the colon and/or following the in vitro faecal fermentation. For instance, the syringaresinol (125.60 mg/kg) was the second most abundant compound measured in the raw BHs, with a high proportion (73.05 mg/kg) remaining in bound form (54.71 mg/kg to 79.90 mg/kg) following the first bioprocessing, and after the second treatment (105.40 mg/kg to 119.70 mg/kg). Enterolactone, which is one of the main microbial metabolites of syringaresinol metabolism produced during the microbial incubations, was undetectable at 0 h and rapidly increased after the 24 h fermentation process. However, there is no obvious difference in the colonic release of enterolactone, due to the large interindividual variation in the capacity of the colonic microbiota in converting plant lignans to enterolactone [27,28] and further into the enterodiols. Given that enterolactone is associated with reduced risk of coronary diseases and colorectal adenoma in humans, the increased intake of syringaresinol-rich food sources such as BHs has the potential to be explored as an enterolignan source for human health. Furthermore, several abovementioned PADs, found in high proportion in bound forms, are considered to have a strong antioxidative property and prevent diseases associated with antioxidative stress, such as cancer, ageing, inflammation, etc. This further supports the use of BH fibre to promote human health and, in general, that the consumption of wholegrain crops promotes health due to their rich phytochemical and fibre content [19]. 

The production of SCFAs plays a major function of the gut microbiota associated with protection from many metabolic diseases, such as obesity, type 2 diabetes, and cardiovascular diseases [29]. The microbial synthesis of SCFAs is mainly mediated by the fermentation of indigestible NSPs. In most situations, soluble NSPs are more readily fermented by gut microbes than the insoluble type, resulting in a rapid increase in SCFA production [30]. Here, the enzymatic bioprocessed EM_1 and EC_2 effectively increased the content of insoluble NSPs, while being incapable of increasing the content of soluble NSPs; as a result, the SCFAs production remains similar between raw, and after EM_1 and EC_2 treatments. In our previous study focusing on the bioprocessing of hemp screenings, EM_1 and EC_2 effectively increased the content of soluble NSPs, but their SCFA production after colonic fermentation was similar because of the high fermentability of NSPs in hemp screenings [31]. Other studies found that combined microwave and enzymatic processing induced an 8% increase in soluble bran fibre, which contributed to doubled butyrate production and almost tripled acetate production [32]. The IVDG pre-digestion also had a limited impact on SCFA production for raw and bioprocessed BHs. Despite the similar SCFA production between groups, the production of each SCFA clearly differed between donor 2 and the other two donors, reflected in the high SD value. Similarly, the microbial metabolism of phytochemicals was also characterised by very high inter-donor variations during faecal incubations.

The donor-dependent production of microbial metabolites (SCFAs and biotransformation of plant metabolites) is most probably associated with the inter-individual variations in microbiota composition [33,34]. Indeed, in donor 1 and 3, both the richness and diversity of microbial communities were very similar and displayed consistent alterations after the addition of raw BHs, which distinguished them from the corresponding changes in donor 2. The addition of raw BHs particularly reduced *B. vulgatus* (OTU 2, 98.81%), *Clostridium fessum* (OTU 6, 98.02%), *Anaerotignum lactatifermentans* (OTU 11, 100%) in donor 2, which was associated with the smaller increment in acetate and total SCFAs than that in donor 1 and 3 [35]. Furthermore, in donor 3, the addition of raw BHs consistently displayed negligible impacts on the community composition at family, genus, and OTU levels, which was associated with the moderate increment in total SCFAs. In donor 1, *Oscillospiraceae bacterium* (OTU 4, 100%) was uniquely enriched with the addition of raw BHs, associated with the highest increment in butyrate and total SCFAs. Accordingly, the different microbiota structures exhibited function redundancy in SCFA production during BH fermentation. In comparison, the microbial conversion of phytochemicals in BHs is likely to involve a multi-species pathway, which has been demonstrated by the fermentation of wheat bran with isolated butyrate-producing bacteria [36]. 

In conclusion, buckwheat hulls are a rich source of dietary fibre, mainly insoluble but fermentable. Moreover, buckwheat hulls are a rich source of bioactive phytochemicals such as protocatechuic acid and syringaresinol, with proven health benefits to humans. The common buckwheat was used as the model substrate in this study; the other varieties are likely to have differing profiles of phytochemicals and nutrient content and could be the scope of further research using a similar approach. The bioprocessing treatments, especially EC_2 treatment, was more efficient in increasing the in vitro upper gastrointestinal release of the plant metabolites from hulls in comparison with the EM_1 treatment. Therefore, this treatment could be successfully used to promote fibre-rich ingredients as sources of bioavailable phytochemicals. The bioprocessing treatments had no effect on the microbial metabolism of the buckwheat hulls components such as dietary fibre fermentation.

These findings provide strong evidence to promote the use of buckwheat hulls as a source of dietary fibre and phytochemicals with proven health benefits, suggesting the necessity to further explore this ingredient for the development of functional foods for promoting metabolic and gut health and ultimately to help diversify and meet dietary recommendations for fibre.

## 4. Materials and Methods

### 4.1. Standards and Reagents

Standards and general laboratory reagents were purchased from Sigma-Aldrich (Gil-lingham, UK) and Fisher Scientific UK Ltd. (Loughborough, UK) or synthesised as described previously [24,37]. 

Buckwheat hulls were purchased on the local market in Aberdeen, Scotland UK. BHs were milled with liquid nitrogen (Spex 6700, Edison, NJ, USA) and the powder was used as matrix for phenolics extraction, bioprocessing treatments, and in vitro fermentation experiment [24].

### 4.2. Bioprocessing of Buckwheat Hulls

The buckwheat hulls bioprocessing was performed as described by Fan et al. [31]. Briefly, seven different bioprocessing samples were distinguished into two main groups, namely first and second bioprocessing. In the first bioprocessing treatment, the enzyme mixtures I (EM_1) were prepared with three commercial carbohydrate-hydrolysing food grade enzymes: 0.01% (*w*/*w*) Fungamyl^®^ 800 L, 0.14% (*w*/*w*) Viscozyme^®^ L (Novozymes Corp., Bagsvaerd, Denmark), 0.36% (*w*/*w*) Depol 740 L, Biocatalysts Ltd., Wales, UK) and Millipore water (pH 6.5). Fermentation bioprocessing (BY_1) was performed by mixing 22% (*w*/*w*) hempseed screenings and 0.27% (*w*/*w*) Baker’s yeast (Sigma-Aldrich, Darmstadt, Germany) with Millipore water. For the EM+BY_1 group, enzyme mixture I was applied along with yeast fermentation. All the mixtures in the first bioprocessing were kept at 20 °C for 20 h in a shaker at 150 rpm. 

In the second bioprocessing, EM_2 used enzyme mixture I. EC_2 was treated with enzyme mixtures II, which were prepared with the combination of enzyme mixture I and 0.01% (*w*/*w*) cellulase from *Trichoderma* sp. (Sigma-Aldrich, Gillingham, UK). Fermentation bioprocessing (BY_2) was continuously performed by mixing 22% (*w*/*w*) hempseed screenings and 0.27% (*w*/*w*) Baker’s yeast. For the EC+BY_2, enzyme mixtures II were applied along with Baker’s yeast fermentation. The second bioprocessing used Millipore water adjusted to pH 5 and contained two phases: an initial incubation at 40 °C for 4 h and then 20 °C for 20 h without mixing. After bioprocessing, these mixtures in triplicate were directly freeze-dried for the subsequent experiments.

### 4.3. Macronutrient Analysis

All analyses were performed as described by Multari et al. [24]. Protein was measured as total nitrogen by the Dumas combustion method using a Vario Max CN analyser, and the nitrogen content was multiplied by 6.25 to generate the protein concentration. Resistant starch and non-starch polysaccharide were measured according to the methods described previously [38,39]. Total fat was determined by the Soxtec method (Soxtec 2050 Auto Fat Extraction System). The amino acid analysis was done by Alta Bioscience Ltd. (Redditch, UK), using ISO/IEC 17025:2017 [40] accredited methodologies.

### 4.4. Extraction of Phenolic Compounds

Phenolic extraction was conducted according to a proposed procedure [37]. Briefly, samples (approx. 0.1 g dry weight; n = 3) were suspended in HCl (0.2 M; 3 mL) and then extracted into ethyl acetate (EtOAc) (5 mL). The extraction was repeated twice again. The combined EtOAc extracts were dried and the residue was dissolved with methanol (0.5 mL), which represented the “free fraction” and was stored in −70 °C prior to LC-MS analysis. 

The remaining aqueous fraction was neutralised and freeze-dried for extraction of ‘bound fraction’. The freeze-dried pellets were suspended in NaOH (3 mL; 1 M) and stirred at room temperature for 4 h under nitrogen. The pH was reduced to 2 with HCl (6 M), and samples were extracted into EtOAc (5 mL). This was repeated twice and processed as described above to obtain ‘alkaline-bound fraction’.

The pH of the aqueous fraction was then brought to 7 and freeze-dried again. The freeze-dried aqueous fractions were suspended in HCl (3 mL; 2 M) and incubated at 95 °C for 30 min. The samples were cooled to room temperature and extracted with EtOAc (5 mL) three times again and processed as described above to have ‘acid-bound fraction’. 

To prepare samples for LC-MS analysis, an aliquot of each type of methanol-dissolved extract prepared above was mixed with Internal standard 1 (IS1) for negative-mode mass spectrometry (13C benzoic acid); and internal standard 2 (IS2) for positive-mode mass spectrometry (2-amino-3,4,7,8-tetramethylimidazo [4,5-ƒ]quinoxaline).

### 4.5. Simulated Gastrointestinal Digestion and Dialysis

In vitro gastrointestinal digestion (IVDG) was performed according to the harmonised protocol developed by the COST INFOGEST network, with slight modifications [31]. The IVDG model consists of an oral phase, a gastric phase, and an intestinal phase. All solutions were preheated to 37 °C prior to use, and all the experimental conditions were performed in a pre-set 37 °C shaking water bath (Grant OLS-200, Cambridge, UK). Briefly, in the oral phase, weighed sample (1 g) in triplicate was mixed with 3 mL of water to create a ‘food-like’ paste. Next, 50% (*v*/*v*) Simulated Salivary Fluid (SSF, pH 7) containing calcium chloride (CaCl_2_) (150 mM) and amylase (150 U/mL) was added to the paste and was allowed to mix for 2 min. Followed by mixing, the sample from the oral phase with the same volume of gastric phase, Simulated Gastric Fluid (SGF, pH 3) containing (16 mg/mL) and CaCl_2_ (0.15 mM). After adjusting to pH 3.0 with 1 M HCl, the sample was incubated for 2 h, stimulating gastric phase. Then the simulated food bolus was exposed to Simulated Intestinal Fluid (SIF, pH 7) at 50% (*v*/*v*) that contained pancreatin (without bile acids) and was incubated for further 3 h after the pH was brought to 7.0 with 1 M NaOH.

The digesta from the IVDG were transferred to a 50 mL falcon tube and centrifuged (3200× *g*, 5 min, 4 °C). The pellet was kept at −20 °C. The supernatant was transferred into a cellulose dialysis membrane (Cheshire biotech, Cheshire, UK) with a molecular weight cut-off of 12–14 kDa and dialysed against a 100-fold volume of denoised water for one hour at room temperature, repeated twice again. The dialysis fluid from outside of dialysis membrane was collected and combined, 30 mL aliquots were sampled from 4.5 L homogeneous dialysis fluid and analysed for metabolites by LC-MS/MS, which represent the small molecules probably absorbed in the small intestine. The digesta retained inside of dialysis membrane was combined with saved cold pellets, which means the part passed to colon. After freeze-drying, the obtained samples named as BH_IVGD were used as substrate for in vitro fermentation experiment. Meanwhile, the raw BHs without IVGD were labelled as BH_Raw in the in vitro fermentation experiment to distinguish from BH_IVGD.

### 4.6. LC-MS Analysis

Liquid chromatography separation of phenolic metabolites was performed on an Agilent 1100 LC-MS system using a Zorbax Eclipse 5 µm, 150 mm × 4 mm column from Agilent Technologies (Wokingham, UK) as described elsewhere [24]. All extracts and dialysed samples prepared above were screened for phenolic acid, flavonoids, and lignan metabolites. Three gradients were used to separate the different categories of metabolites, and the mobile-phase solvents in each case were water containing 0.1% acetic acid (A) and acetonitrile containing 0.1% acetic acid (B). Method 1: 10–55% B (45 min), 55–95% B (5 min), 95–95% B (2 min), 95–10% B (1 min), 10–10% B (7 min); Method 2: 10–95% B (40 min), 95–95% B (3 min), 95–15% B (1 min), 15–15% B (6 min); Method 3: 40–90% B (13 min), 90–90% B (1 min), 90–40% B (1 min), 40–40% B (9 min). In all cases, the flow rate was 300 µL/min with an injection volume of 5 µL. The LC eluent was directed, without splitting, into an ABI 3200 triple-quadrupole mass spectrometer (Applied Biosystems, Warrington, UK) fitted with a turbo ion spray source. The mass spectrometer was run in negative ion mode with the following source settings: ion spray voltage, −4500 V; source temperature, 400 °C; gases 1 and 2 set at 15 and 40 (units), respectively; and the curtain gas set to 10 (units). All the metabolites were quantified using multiple reaction monitoring. Standard solutions (10 ng/µL) for all analytes were prepared and pumped directly via a syringe pump. The ion transitions for each of the analytes were determined based on their molecular ion and a strong fragment ion. For several categories of compounds, it was inevitable that their molecular ion and fragment ion would be the same, but this was overcome by their different elution times.

### 4.7. In Vitro Colonic Fermentation

#### 4.7.1. Human Faecal Incubation

BH_Raw and BH_IVGD were used as substrates for in vitro fermentation experiment. Weigh 20 ± 1 mg materials in six duplicates into pre-weighed Hungate tubes sealed with butyl rubber stoppers and screw caps (Bellco Glass, Shrewsbury, UK). Under an anaerobic condition CO_2_ maintained, 7.5 mL M2 basal medium (containing no soluble starch, glucose, and cellobiose, M2SGC) were added into those Hungate tubes, following by subsequent autoclaving and cooling to room temperature. The faecal slurry was achieved by anaerobically mixing 2 g of fresh faeces (faecal samples were collected from 3 different donors, named donor 1, 2, and 3) with 8 mL of anaerobic 50 mM phosphate buffer containing 0.05% cysteine and vortex mixing. Three tubes of six duplicates were inoculated with the 100 µL faecal slurry under CO_2_ and incubated at 37 °C, while three control tubes were added with 100 µL sterile buffer. Samples for microbial metabolites analysis were recovered at 0 h, 24 h, 48 h, and 72 h post-inoculation. Samples recovered at 0 h and 72 h were also used for SCFA analysis. Samples recovered at 72 h were also used for DNA extraction.

#### 4.7.2. Microbial Metabolites Analysis

The incubated fluid was mixed with a specific volume of methanol and internal standard solution (1:2:2, *v*/*v*/*v*), and centrifuged at 12,000× *g* for 10 min. The supernatant was used for LC-MS/MS injection (see Section 4.5).

#### 4.7.3. Short Chain Fatty Acids (SCFAs) Analysis

SCFA formation was measured in culture supernatants by gas chromatography as described previously [41]. Following conversion to tert-butyldimethylsilyl derivatives, 1 μL of sample was analysed using a Hewlett-Packard (Palo Alto, CA, USA) gas chromatograph fitted with a fused silica capillary column with helium as a carrier gas. The SCFA concentrations were calculated from the relative response factor with respect to the internal standard (two-ethyl butyrate).

#### 4.7.4. Microbial Analysis

The fermentation samples collected at 72 h were centrifuged at 10,000× *g* for 10 min to pellet bacterial cells, and the pellets were used to extract microbial DNA using the FastDNA Spin kit (MP Biomedicals, Illkirch, France). The concentration and purity of the extracted DNA were measured with a Qubit 2.0 fluorometer (Life technologies, Carlsbad, CA, USA). The V4 region of bacterial 16S rRNA genes was amplified with polymerase chain reaction (PCR) using the barcoded fusion primers MiSeq-515F (5′-AATGATACGGCGACCACCGAGATCTACACGCT-barcode-TATGGTAATTGTGTGYCAGCMGCCGCGGTAA-3′) and MiSeq-806R (5′-CAAGCAGAAGACGGCATACGAGATAGTCAGCCAGCCGGACTACNVGGGTWTCTAAT-3′), which also contain adaptors for downstream Illumina MiSeq sequencing and a unique (12-base) barcoded forward primer. Sequencing was carried out on an Illumina MiSeq machine, using 2 × 300 bp read length, at the Centre for Genome-Enabled Biology and Medicine (CGEBM) in University of Aberdeen. Sequence data have been deposited in the European Nucleotide Archive and are available under study accession number PRJEB66317 and sample accession numbers ERS16413777 to ERS16413782.

The sequencing data were analysed using the mothur v.1.39.5 software, as described previously. In brief, forward and reverse reads were assembled into paired read contigs (in total 901,842 sequences). Resulting contigs that were shorter than 200 bp, longer than 460 bp, contained ambiguous bases, or contained homopolymeric stretches of longer than 7 bases, were all removed. Unique sequences were then grouped together and aligned against the SILVA reference database. Preclustering (diffs = 3) was carried out to reduce the impact of sequencing errors and the OTUs were generated at a 97% similarity cut-off level. All samples were rarefied to 93,500 reads to ensure equal sequencing depth for all comparisons. Taxonomic classifications for each OTU from phylum to genus level were assigned using the RDP reference database (release 18). To assign species-level classifications, representative sequences for each OTU were searched against the NCBI Nucleotide database using MegaBLAST. The observed out (Sob), Chao 1, Shannon diversity, and inverse Simpson diversity indexes were used to calculate bacterial diversity per sample.

### 4.8. Statistical Analysis

Significance differences between groups were tested by one-way ANOVA and Turkey’s multiple comparisons, with a significance threshold of * *p* < 0.05 and ** *p* < 0.01. All the analyses were performed in triplicate, and results were presented as the mean ± S.D. The microbial metabolites profiles measured by LC-MS/MS were determined by principal component analysis (PCA), unit variance (UV)-scaled using SIMCA 14.1 (Umetrics, Cambridge, UK), and heatmaps (log10 transformed) were visualised using R 4.0.3.

## Figures and Tables

**Table 1:** Nutritional composition and amino acids composition of buckwheat hulls (% *w*/*w*). The values are expressed as g/100 g dry weight ± SD (standard deviation).

Nutrient Composition	Content (%)	Amino Acid Composition			
		EAA	Content (%)	Non-EAA	Content (%)
Moisture	10.8 ± 0.0	Leucine	0.l9	Glutamic acid	0.33
Ash	1.7 ± 0.0	Tryptophan	0.15	Aspartic acid	0.25
Protein	4.05 ± 0.05	Threonine	0.l4	Cysteic acid	0.23
Total fat	0.1 ± 0.0	Valine	0.13	Glycine	0.16
Total starch	2.55 ± 0.0	Phenylalanine	0.11	Serine	0.15
Resistant starch	n.d.	Histidine	0.11	Proline	0.14
Total NSPs		Isoleucine	0.11	Alanine	0.13
-Insoluble	31.1 ± 0.2	Lysine	0.09	Arginine	0.1
-Soluble	0.30 ± 0.05	Methionine	0.02	Tyrosine	0.04
				Cystine	n.d.

Where NSPs: non-starch polysaccharides; n.d. = not detected (i.e., below the detection level); EAAs and non-EAAs are the essential amino acids and non-essential amino acids, respectively.

**Table 2:** Non-starch polysaccharides (NSPs) in the raw and bioprocessed buckwheat hulls (BHs). Values were expressed as the mean ± S.D., and the total value was the sum of all analysed monosaccharide units (n = 3). A *t*-test with FDR correction was used for group comparison between Raw and EM_1, EM_2 or EC_2. (*) *p* < 0.05 and (**) *p* < 0.01.

NSPs	Raw	First Bioprocess			Second Bioprocess			
		EM_1	BY_1	EM+BY_1	EM_2	EC_2	BY_2	EC+BY_2
**Insoluble (%)**								
Xylose	15.78 ± 0.27	19.73 ± 0.65 **	19.50 ± 1.82	18.94 ± 0.07	20.51 ± 0.23 **	20.37 ± 0.33 **	18.26 ± 0.63	18.72 ± 0.44
Glucose	9.67 ± 0.34	20.07 ± 1.35 **	16.83 ± 0.62	14.79 ± 0.18	10.89 ± 1.17	11.04 ± 1.28	10.27 ± 0.56	10.39 ± 0.82
Arabinose	0.72 ± 0.01	0.82 ± 0.04 **	0.82 ± 0.00	0.80 ± 0.02	0.83 ± 0.01 **	0.84 ± 0.01 **	0.78 ± 0.04	0.76 ± 0.01
Galactose	0.61 ± 0.01	0.67 ± 0.03 **	0.63 ± 0.04	0.65 ± 0.00	0.70 ± 0.01 **	0.71 ± 0.00 **	0.67 ± 0.03	0.66 ± 0.01
Rhamnose	0.33 ± 0.01	0.43 ± 0.03 **	0.40 ± 0.02	0.39 ± 0.01	0.37 ± 0.02	0.38 ± 0.00 *	0.37 ± 0.02	0.37 ± 0.01
Mannose	0.24 ± 0.01	0.30 ± 0.04 **	0.42 ± 0.03	0.40 ± 0.01	0.26 ± 0.01	0.27 ± 0.00	0.33 ± 0.02	0.33 ± 0.02
Fucose	0.07 ± 0.00	0.07 ± 0.00 **	0.10 ± 0.03	0.07 ± 0.01	0.08 ± 0.00 **	0.08 ± 0.00 **	0.07 ± 0.00	0.08 ± 0.01
Uronic Acid	3.68 ± 0.11	4.31 ± 0.01	3.90 ± 0.12	4.06 ± 0.02	4.05 ± 0.18	3.75 ± 0.56	3.77 ± 0.07	4.34 ± 0.53
**Total**	**31.09 ± 0.22**	46.41 ± 2.12 **	**42.60 ± 1.14**	**40.09 ± 0.25**	37.68 ± 1.26 **	37.43 ± 0.68 **	**34.51 ± 1.21**	**35.64 ± 0.57**
**Soluble (%)**								
Xylose	0.06 ± 0.01	0.06 ± 0.02	0.06 ± 0.02	0.05 ± 0.03	0.02 ± 0.02	0.04 ± 0.04	0.04 ± 0.02	0.01 ± 0.02
Glucose	0.09 ± 0.01	0.09 ± 0.03	0.07 ± 0.01	0.09 ± 0.04	0.04 ± 0.02	0.06 ± 0.04	0.07 ± 0.02	0.03 ± 0.00
Arabinose	0.02 ± 0.00	0.03 ± 0.00 *	0.04 ± 0.01	0.03 ± 0.00	0.02 ± 0.00	0.03 ± 0.01 *	0.03 ± 0.01	0.03 ± 0.01
Galactose	0.02 ± 0.00	0.00 ± 0.00	0.04 ± 0.01	0.00 ± 0.00	0.00 ± 0.00	0.01 ± 0.01	0.03 ± 0.01	0.01 ± 0.03
Rhamnose	0.00 ± 0.00	0.00 ± 0.00	0.03 ± 0.00	0.02 ± 0.02	0.00 ± 0.00	0.00 ± 0.00	0.00 ± 0.00	0.01 ± 0.02
Mannose	0.00 ± 0.00	0.06 ± 0.00 **	0.10 ± 0.00	0.08 ± 0.01	0.00 ± 0.00	0.00 ± 0.00	0.00 ± 0.00	0.01 ± 0.02
Fucose	0.03 ± 0.00	0.05 ± 0.01	0.04 ± 0.00	0.04 ± 0.00	0.03 ± 0.02	0.04 ± 0.01	0.04 ± 0.00	0.03 ± 0.00
Uronic Acid	0.09 ± 0.03	0.02 ± 0.00 **	0.15 ± 0.03	0.04 ± 0.01	0.01 ± 0.00 **	0.01 ± 0.00 **	0.09 ± 0.03	0.01 ± 0.00
**Total**	**0.30 ± 0.05**	**0.31 ± 0.06**	**0.54 ± 0.01**	**0.34 ± 0.07**	**0.12 ± 0.04**	**0.18 ± 0.10**	**0.31 ± 0.09**	**0.15 ± 0.08**

**Table 3:** The composition of metabolites (measured by targeted LC-MS/MS) in the fraction resulting after in vitro digestion of the raw and bioprocessed (EM_1 and EC_2) buckwheat hulls. Values were expressed with mean of three replicates and standard deviation.

Plant Metabolites (mg/kg)	Raw		EM_1		EC_2	
	Free Fraction Content (Initial)	Content after In Vitro Digestion	Free Fraction Content (Initial)	Content after In Vitro Digestion	Free Fraction Content (Initial)	Content after In Vitro Digestion
protocatechuic acid	184.81 ± 21.00	300.18 ± 3.46	185.37 ± 8.24	268.99 ± 1.59 **	193.21 ± 3.07	346.67 ± 6.28 **
protocatechualdehyde	37.16 ± 1.54	29.04 ± 0.69	35.80 ± 2.91	30.90 ± 1.56	36.18 ± 1.63	33.11 ± 0.61 **
3, 4, 5-trihydroxybenzoic acid	28.50 ± 1.67	1.10 ± 0.18	25.65 ± 1.27	1.17 ± 0.39	57.38 ± 1.53	4.82 ± 0.33 **
vanillin	11.40 ± 0.78	5.07 ± 0.17	3.11 ± 0.08	10.08 ± 0.61 **	14.57 ± 1.20	8.89 ± 0.75 *
*p*-hydroxybenzoic acid	9.55 ± 0.43	19.85 ± 0.09	14.16 ± 0.81	21.98 ± 0.13 **	11.30 ± 0.95	26.36 ± 0.32 **
3, 5-dimethoxy-4-hydroxybenzaldehyde	6.85 ± 0.46	6.42 ± 0.09	5.49 ± 0.41	7.08 ± 0.45	8.57 ± 0.39	7.09 ± 0.49
vanillic acid	5.67 ± 0.23	9.54 ± 0.10	10.99 ± 0.94	9.89 ± 0.34	6.47 ± 0.19	12.65 ± 0.29 **
*p*-hydroxybenzaldehyde	4.97 ± 0.39	4.24 ± 0.08	2.14 ± 0.18	8.32 ± 0.55 *	8.03 ± 0.57	8.55 ± 0.17 **
salicylic acid	4.47 ± 0.32	6.03 ± 0.14	4.10 ± 0.20	5.27 ± 0.27 *	5.57 ± 1.10	7.36 ± 0.26 **
chlorogenic acid	2.32 ± 0.25	1.41 ± 0.08	2.93 ± 0.49	0.46 ± 0.05 **	1.85 ± 0.20	0.40 ± 0.06 **
3, 5-dimethoxy-4-hydroxybenzoic acid	2.26 ± 0.13	3.64 ± 0.19	2.77 ± 0.18	3.49 ± 0.06	2.68 ± 0.22	4.17 ± 0.18 *
3-hydroxymandelic acid	2.00 ± 0.81	8.71 ± 0.49	2.61 ± 0.13	13.17 ± 0.02 **	n.d.	13.89 ± 0.28 **
2-hydroxybenzyl alcohol	1.91 ± 0.13	1.03 ± 0.05	2.01 ± 0.13	1.15 ± 0.14	1.33 ± 0.15	1.39 ± 0.10 *
*p*-coumaric acid	1.46 ± 0.11	7.10 ± 0.32	1.56 ± 0.11	12.26 ± 0.36 **	8.81 ± 0.82	14.48 ± 0.36 **
phenylacetic acid	1.12 ± 0.06	6.06 ± 0.60	2.94 ± 0.23	7.57 ± 0.66	n.d.	7.45 ± 0.31
ferulic acid	0.69 ± 0.02	1.59 ± 0.04	1.58 ± 0.37	2.59 ± 0.16 *	3.01 ± 0.84	3.95 ± 0.23 **
3, 4-dihydroxyphenylacetic acid	n.d.	7.47 ± 0.76	n.d.	7.44 ± 0.49	6.98 ± 0.69	10.73 ± 0.43 *
4-hydroxyphenylacetic acid	n.d.	4.17 ± 0.11	n.d.	6.82 ± 0.45 *	n.d.	9.66 ± 0.65 **
caffeic acid	n.d.	0.80 ± 0.03	0.26 ± 0.04	1.13 ± 0.03 **	3.89 ± 0.36	1.95 ± 0.11 **
2, 3-dihydroxybenzoic acid	n.d.	0.43 ± 0.04	0.28 ± 0.24	0.59 ± 0.02 *	1.87 ± 0.10	2.32 ± 0.08 **

n.d. means not detected (i.e., below the detection level). The total content (initial) represents the sum of the free, alkaline, and acid forms of individual metabolites measured by LC-MS/MS (Table S1). A *t*-test with FDR correction was used for group comparison between Raw and EM_1 as well as Raw and EC_2. (*) *p* < 0.05 and (**) *p* < 0.01.

## Data Availability

The raw sequencing data for all samples have been deposited into NCBI Sequencing Read Archive under accession number PRJNA1025115.

## Supplementary Materials

The supporting information can be downloaded at: https://www.mdpi.com/article/10.3390/ijms242216310/s1.
