# Supplementary material for: Buckwheat (Fagopyrum esculentum) Hulls Are a Rich Source of Fermentable Dietary Fibre and Bioactive Phytochemicals
Source: Int J Mol Sci. 2023 Nov 14;24(22):16310. doi: 10.3390/ijms242216310 (PMC10671810; doi:10.3390/ijms242216310)
Supplement: Supplementary file 1 [file ijms-24-16310-s001.zip › Fig S1 and Fig S2 BW hulls Int J Mol Sci 12 nov .pdf]

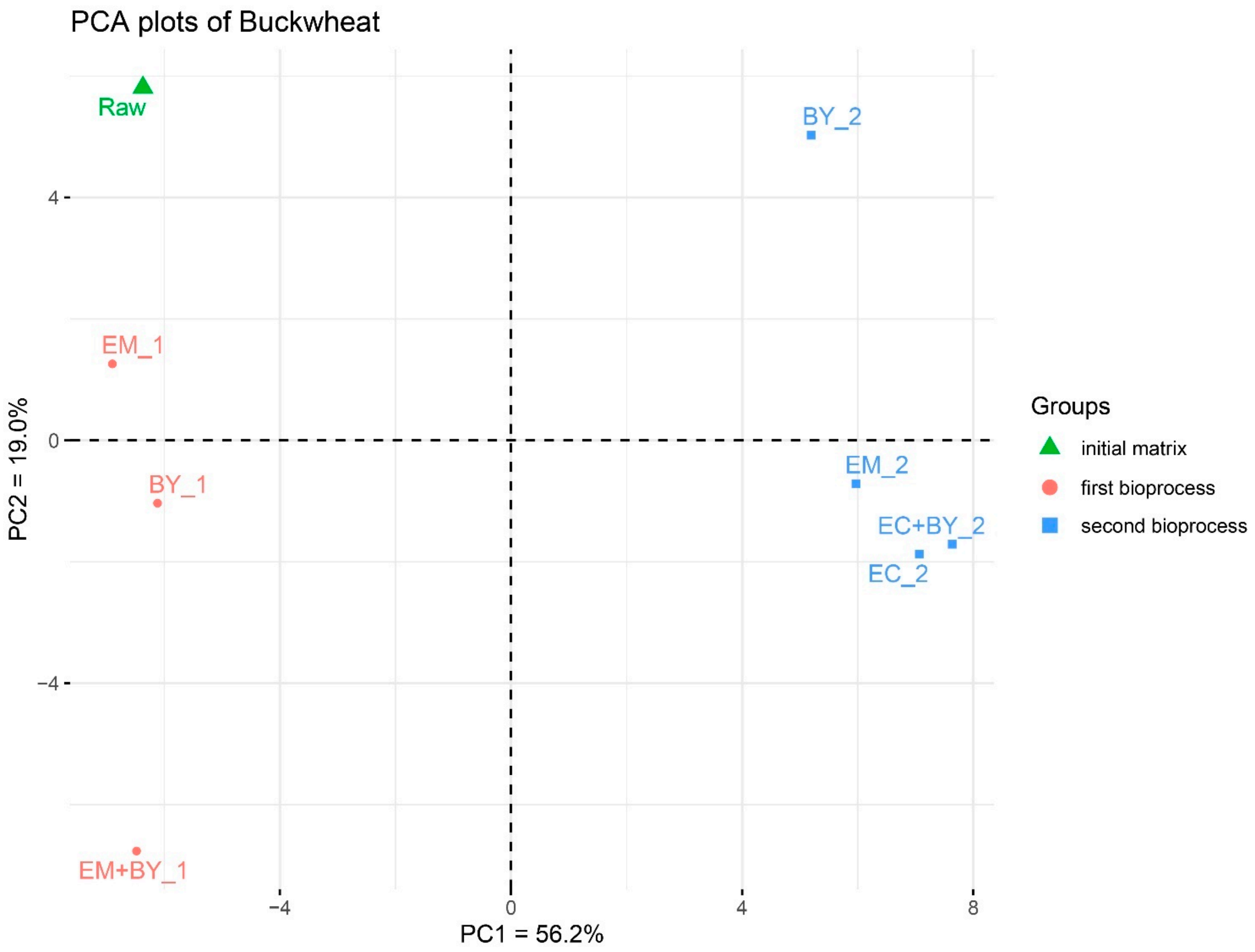

Figure S1. Principal component analysis showing discrimination between raw, first bioprocessed, and second bioprocessed BH based on the total content (the sum of free, alkaline, and acid fractions) for all the plant metabolites measured by LC-MS/MS.

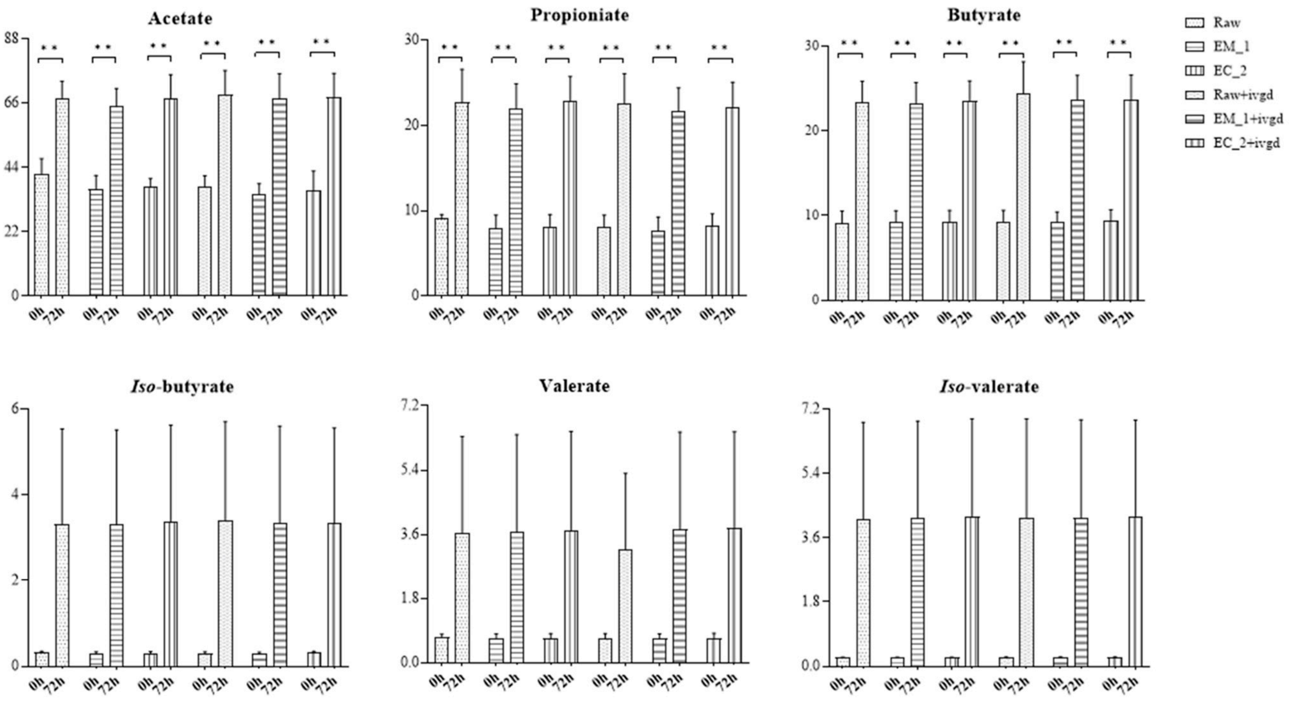

Donor	Acetate	Propionate	iso-Butyrate	Butyrate	iso-Valerate	Valerate
	mM					
1	35.4±5.6	10.83±1.45	1.65±1.47	12.16±1.83	2.45±2.2	3.73±2.74
2	34.6±11.1	11.17±3.74	0.99±2.55	12.29±1.88	1.34±3.41	0.71±0.21
3	35.01±4.42	9.09±1.27	0.46±0.1	13.31±3.41	0.65±0.16	2.6±0.7

Figure S2. Short-chain fatty acids (SCFA) content (mM) at start point (0 h) and end point (72 h) of fermentation process, expressed as average ± SD (n = 3). The raw and enzyme-treated buckwheat hulls (EM_1 and EC_2), as well as their IVDG-predigested samples, were inoculated with faecal slurries from each of the three donors (Donor 1, 2 and 3). Values were calculated by subtracting the value of 0 h from the value of the fermented sample at 72 h for each sample. Values for faecal control short chain fatty acids formed during 72h incubation (minus 0h values) with mixed faecal microbiota in basal growth medium are presented in the bottom of the plot. Significance differences were (*) $p < 0.05$ and (**) $p < 0.01$ as compared to the 0h in each group using the Two-way ANOVA for multiple comparisons.
